# Cecal Microbial Succession and Its Apparent Association with Nutrient Metabolism in Broiler Chickens

**DOI:** 10.1128/msphere.00614-22

**Published:** 2023-04-05

**Authors:** Zhenchen Yin, Shuyun Ji, Jiantao Yang, Wei Guo, Yulong Li, Zhouzheng Ren, Xiaojun Yang

**Affiliations:** a College of Animal Science and Technology, Northwest A&F University, Yangling, Shaanxi, People’s Republic of China; b School of Basic Medical Sciences, Shaanxi University of Chinese Medicine, Xianyang, Shaanxi, People’s Republic of China; University of Michigan

**Keywords:** broiler chickens, cecal microbiota, temporal colonization, developmental stage, nutrient metabolism

## Abstract

The chicken gut microbiota plays an influential role in nutrient absorption and metabolism. A clear picture of microbiota succession can enhance host nutrition and disease resistance. This study investigated the cecal microbiota succession of broilers between 3 and 42 days after hatching using 16S rRNA gene sequencing and analyzed its potential association with intestinal nutrient metabolism. Microbiota structure differed significantly at different time points depending on the microbiota alpha-diversity or beta-diversity. *Proteobacteria* and *Bacteroidetes* promoted succession on days 3 to 7 and days 28 to 35, respectively. *Firmicutes* and *Tenericutes* maintained homeostasis on days 7 to 28 and days 35 to 42. *Shigella*, [*Ruminococcus*], *Erysipelotrichaceae_Clostridium*, and *Coprobacillus* promoted succession on days 3 to 7; *Faecalibacterium* modified microbial composition on days 7 to 14; *Faecalibacterium* and *Bacteroides* regulated microbial structure from days 21 to 28. The microbiota structure was relatively stable on days 14 to 21 and days 28 to 35. Spearman’s correlation analysis indicated a positive correlation between *Lactobacillus* and villus height and crypt depth (*P < *0.01). *Faecalibacterium* and *Shigella* were correlated with propionate, butyrate, and valerate concentrations (*P < *0.01). *Ruminococcus* was correlated with sodium-glucose cotransporters 1 and cationic amino acid transporter 1 expression (*P < *0.05). *Erysipelotrichaceae_Clostridium* and *Shigella* were positively correlated with serum levels of total cholesterol, tryglucerides, and high- and low-density lipoprotein cholesterol (*P < *0.01). *Bacteroides*, *Parabacteroides*, *Lactobacillus*, and *Shigella* were correlated with serum VB6 levels (*P < *0.01). *Bacteroides*, *Erysipelotrichaceae_Clostridium*, and *Coprobacillus* were correlated with the moisture content of cecal contents (*P < *0.05). The identification of the microbiota in correlation with nutrient metabolism will promote microbial nutrition through microbiota intervention or nutritional regulation.

**IMPORTANCE** The poultry industry has become a global leader in livestock farming over the past few decades. Poultry production has a large consumer market as an integrated industry producing high-protein foods. Establishing the association between microbiota and nutrient metabolism processes provides fresh insights for precise nutrient regulation. This study aimed to describe the development of cecal microbiota in broiler chickens throughout the production cycle and to assess the correlation of nutrient metabolism phenotypes with temporal changes in the microbiota. The results suggested that changes in cecal microbes with age partly explain changes in gut nutrient metabolic processes, and numerous microbes were significantly associated with the processes. Therefore, this study attempts to further find efficient ways of improving poultry production. One is to promote nutrient metabolism by identifying potential candidates for probiotics, and another is to foster the dominant colonization of the microbiota by regulating nutrient metabolism.

## INTRODUCTION

Since the 20th century, the poultry industry has provided numerous high-quality proteins for human nutrition (1), and a steady supply of chicken meat contributes greatly to global food security and stock development. Nutrition is a key component of achieving high-efficiency broiler production, and gastrointestinal microbiology and its metabolic activities are also fundamental factors affecting poultry productivity (2). The microbiota acts as a “metabolic organ” and plays an essential part in the regulation of host physiology. Various physiological functions of the host were mediated by the structural components of microbiota or their metabolites, and these structural components will also be widely affected by nutrients (3). Therefore, elucidating the composition and metabolic functions of the microbiota, as well as the interaction between the microbiota and nutrient metabolic processes, will provide new insights into optimizing the intestinal microecological health and nutritional efficiency of poultry.

The microbiota has various regulatory functions, including host development and nutrition (4), digestive performance (5), intestinal physiology (6), and intestinal immune homeostasis (7). The cecum, the intestinal segment with the greatest microbiota density in broiler chickens (8), can supply more than 10% of the host energy demand. Cecum villi and microvilli transport nutrients such as sugars and amino acids (9). The cecum also contributes to the absorption of water and electrolytes and nitrogenous circulation (10). Cecal microbes play several probiotic roles, including the secretion of active enzymes to deconstruct nonstarch polysaccharides and the fermentation of undigested carbohydrates to produce short-chain fatty acids (SCFAs) (11, 12). Furthermore, it reduces the viscosity of chyme in the intestinal lumen and provides the host with B vitamins (13).

The intestinal microbiota in poultry is changing dynamically, and its diversity and richness will be affected by breed, diet, feeding conditions, and the environment (14). Therefore, elucidating the developmental dynamics of the cecal microbiota will help optimize the microbial composition or timing of nutrition intervention. However, there is no general agreement on the impact of age on the structure of the cecal microbiota of broiler chickens in the relevant studies (15–17). Furthermore, identifying microbiota biomarkers at different developmental stages facilitates the better selection and use of probiotics to meet the needs of different physiological conditions (18–21). For example, promoting the colonization of SCFA-producing probiotics may promote the absorption of nutrients, thereby affecting the metabolic capacity of the host (22, 23). Related studies have also attempted to identify intestinal microbes associated with feed efficiency and phenotypic traits in broiler chickens and laying hens (24–27). However, the microbiota taxa identified so far have different results and poor phenotypic reproducibility.

Therefore, this study aimed to use 16S rRNA gene sequencing to characterize the age-associated dynamic changes in cecal microbiota in broiler chickens and to explore whether there is a correlation between the changes in cecal microbiota and gut nutrient metabolism over time. This study contributes to a better understanding of the baseline development of the gut microbiota in broiler chickens and may lead to the discovery of potential probiotic candidates for precision nutrition or the use of nutritional strategies to promote intestinal microecological balance.

## RESULTS

### The pattern of microbiota succession.

**FIG 1:**
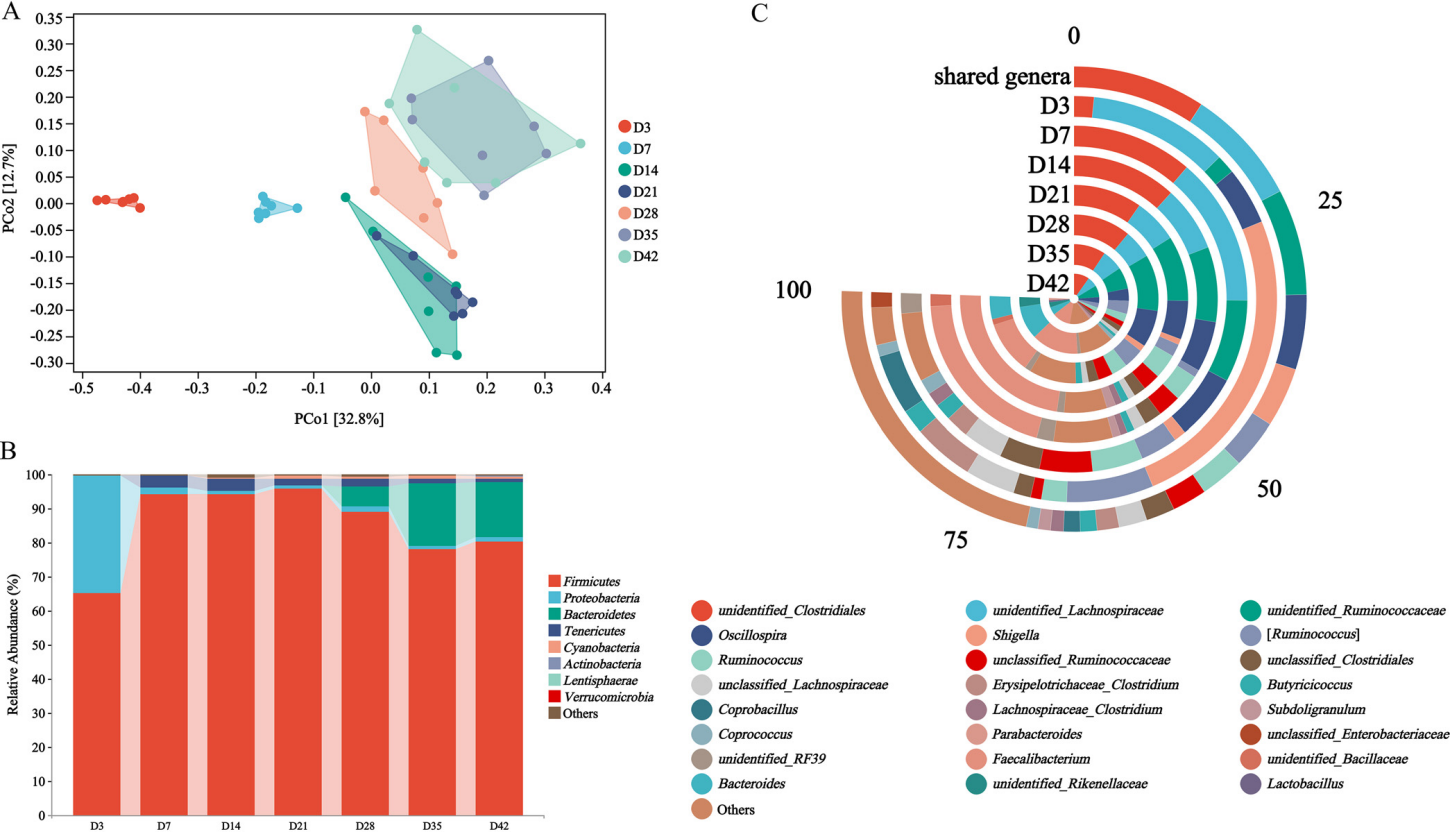
Succession pattern of bacterial taxa in the ceca of broiler chickens. (A) PCoA plots based on the weighted UniFrac distance. (B) Phylum level microbial composition. (C) Shared genera and dominant genera with age.

Overall, the rarefaction curves of all samples supported the adequacy of the sampling efforts (see Fig. S1A in the supplemental material). To evaluate the differences in microbiota diversity, the alpha-diversity was estimated using the index of Chao1, Shannon, Good’s coverage, and Pielou’s evenness (see Fig. S1B). Shannon and Pielou’s evenness index revealed significant changes in species richness and evenness in the cecum with age (see Fig. S1B), and the indices for days 7, 28, and 42 increased significantly in comparison to that for day 3 (*P < *0.01). The Chao1 index of day 3 was significantly lower than that of day 7 (*P < *0.05). Principal coordinate analysis (PCoA) plots based on the weighted UniFrac distance showed the beta-diversity of samples (Fig. 1A). There was very high intragroup similarity on days 3 and 7, and the difference between day 3 and day 7 and other time points was evident (Fig. 1A). From day 14, the gap was decreased between groups.

10.1128/msphere.00614-22.1 FIG S1 Alpha-diversity of microbiota communities in the cecum of broiler chickens. (A) Rarefaction curves of observed species. (B) Index of Chao1, Shannon, Good’s coverage, and Pielou’s evenness. Download FIG S1, TIF file, 8.3 MB. Copyright © 2023 Yin et al. 2023 Yin et al. https://creativecommons.org/licenses/by/4.0/ This content is distributed under the terms of the Creative Commons Attribution 4.0 International license.

At the phylum level, days 3 to 7 represented the rapid developmental stages of the microbiota structure (Fig. 1B). *Firmicutes* (*P < *0.05 versus days 7, 14, 21, and 28) and *Proteobacteria* (*P < *0.05 versus days 14, 21, 28, and 35) were dominantly colonized on day 3, and the microbiota composition was significantly different from other time points (Fig. 1B). However, the relative abundance of *Proteobacteria* decreased on day 7, while the relative abundance of *Firmicutes* continued to increase, followed by *Tenericutes* (Fig. 1B). Between day 7 and day 21, this was the first relatively stable stage. *Bacteroidetes* increased from days 28 to 35 and became the second dominant phylum (Fig. 1B). Days 35 to 42 constitute the second period of relative stability. The *P* values of pairwise comparisons between time points are summarized in Table S1.

10.1128/msphere.00614-22.5 TABLE S1 Summary of *P* values of phylum between any two time points. Download Table S1, PDF file, 0.08 MB. Copyright © 2023 Yin et al. 2023 Yin et al. https://creativecommons.org/licenses/by/4.0/ This content is distributed under the terms of the Creative Commons Attribution 4.0 International license.

### The composition and classification of dominant genera varied with age.

The top 20 genera of seven time points are shown in Fig. S2. The relative abundance of *Faecalibacterium* was lower on days 3 to 7 and higher on days 14 to 21 (*P < *0.01; see also Fig. S2). The relative abundance of *Bacteroides*, *Subdoligranum*, *Lactobacillus*, *Parabacteroides*, *Clostridium*, and *Alistipes* was lowest on day 3 and highest on days 35 or 42 (*P < *0.05; see also Fig. S2). Moreover, the succession of *Blautia* and *cc_115* was similar to the results presented above, but the relative abundance has not changed significantly (see Fig. S2). The *P* values of pairwise comparisons between time points are summarized in Table S2.

10.1128/msphere.00614-22.2 FIG S2 Genus level microbial composition. Download FIG S2, TIF file, 2.5 MB. Copyright © 2023 Yin et al. 2023 Yin et al. https://creativecommons.org/licenses/by/4.0/ This content is distributed under the terms of the Creative Commons Attribution 4.0 International license.

10.1128/msphere.00614-22.6 TABLE S2 Summary of *P* values of genus between any two time points. Download Table S2, PDF file, 0.09 MB. Copyright © 2023 Yin et al. 2023 Yin et al. https://creativecommons.org/licenses/by/4.0/ This content is distributed under the terms of the Creative Commons Attribution 4.0 International license.

To dissect the commonalities and individualities of microbiota succession, shared genera and dominant genera were analyzed (Fig. 1C). The shared genera here have been defined as those which colonized at all time points and had a mean relative abundance above 1% at each time point. The dominant genera were selected from the top 15 at each time point. The main shared genera were *unidentified_Clostridiales*, *unidentified_Lachnospiraceae*, and *unidentified_Ruminococcaceae*, with a relative overall abundance of approximately 32% (Fig. 1C). Other shared genera, such as *Shigella*, [*Ruminococcus*], *unclassified_Lachnospiraceae*, and *Erysipelotrichaceae_Clostridium* were colonized primarily on day 3 (*P < *0.01).

On day 3, the dominant genera were mainly *Shigella*, *unidentified_Lachnospiraceae*, [*Ruminococcus*], and *Erysipelotrichaceae_Clostridium*. From day 7, the relative abundance of *Shigella*, [*Ruminococcus*], *Erysipelotrichaceae_Clostridium*, and *Coprobacillus* decreased appreciably, and changes in relative abundance were greater than at other time points. On day 7, the dominant genera were mainly *unidentified_Lachnospiraceae*, *unidentified_Clostridiales*, and *unidentified_Ruminococcaceae*. The dominant of day 7 was very different from day 3, in particular for *Shigella*, whose relative abundance decreased from 32.92 to 1.49% (Fig. 1C). Compared to day 7, the discernible difference of day 14 was the dominant colonization of *Faecalibacterium*, whose relative abundance increased from zero to 26.44%.

The composition and classification of the dominant genera on day 21 continued with the basic characteristics on day 14 (Fig. 1C). This indicates that days 14 to 21 represented a period of relative stability. The predominant genera were *Faecalibacterium*, *unidentified_Clostridiales*, and *unidentified_Ruminococcaceae* on day 21 (Fig. 1C). The relative abundance of *Faecalibacterium* reached 30.60% on day 21 was noted and became the most dominant genus. Although the composition of microbiota on day 28 was similar to day 21, the relative abundance of the main dominant genera (such as *unidentified_Clostridiales*, *unidentified_Ruminococcaceae*, and *Oscillospira*) has increased, except for *Faecalibacterium* (from 30.59 to 13.42%). Another change was that *Bacteroides* increased from almost zero to 5.86% (Fig. 1C).

The composition of day 35 continued the basic composition on day 28. *Bacteroides* and *unclassified_Rikenellaceae*, with relative abundance close to zero on days 3 to 21 (Fig. 1C). The richness of the genera was further improved on day 42. For instance, the relative abundance of *unidentified_Clostridiales*, *unidentified_Ruminococcaceae*, and *unidentified_Lachnospiraceae* increased slightly from day 35 (Fig. 1C). In addition, *Subdoligranulum*, *Lachnospiraceae_Clostridium*, *Lactobacillus*, and *Parabacteroides* were predominant on day 42 (Fig. 1C).

### Microbiome colonization model at different taxonomic levels.

**FIG 2:**
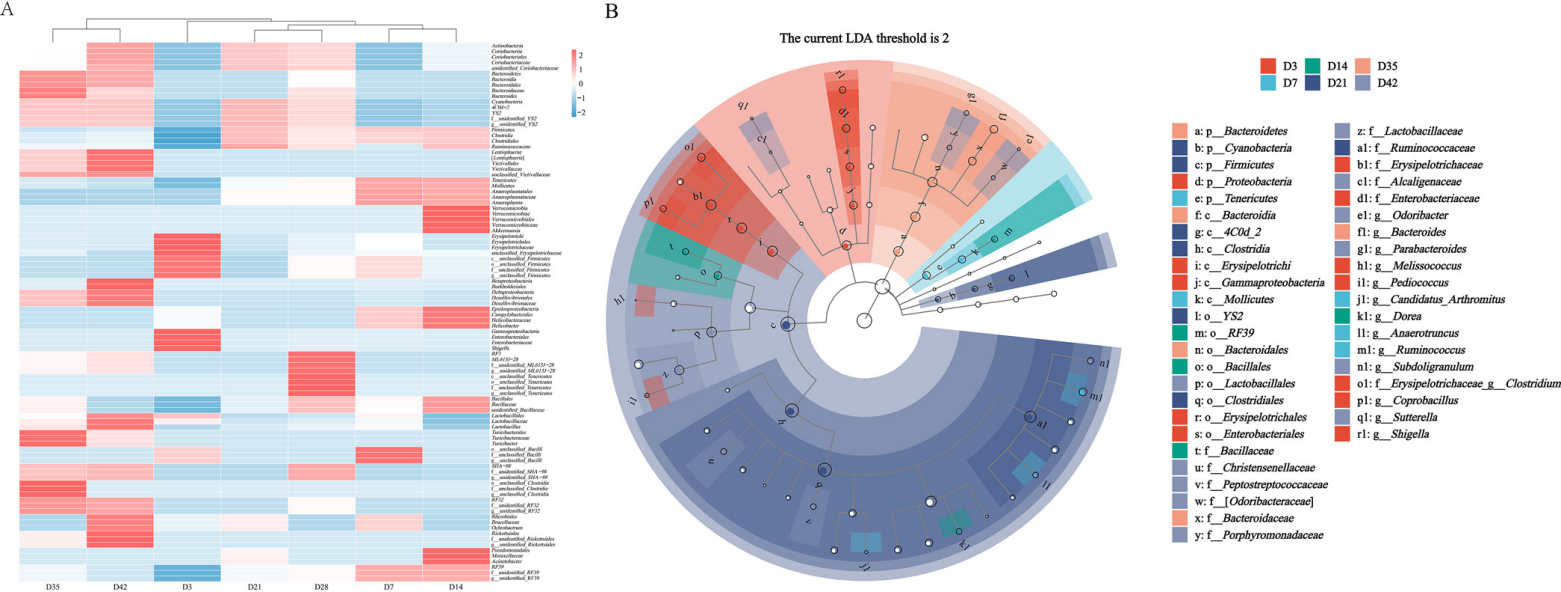
Pattern of microbiota colonization in various taxonomic levels. (A) Microbial taxa with the same change trends in relative abundance with age (two or more levels are the same at phylum, class, order, family, and genus levels). The heatmap color (blue to red, corresponding to low to high) represents the row z-score of the relative abundance values. The *y* axis was clustered according to the microbiota with taxonomic affiliation. (B) LEfSe-derived taxonomic cladogram. The default parameters were LDA score >2 and *P < *0.05.

According to the changes in the relative abundance of microbial groups at different taxonomic levels with age, the microbial taxa with the same relative abundance change trends (here refers to the increase and decrease in abundance were consistent at all-time points) were screened out (Fig. 2A). Taking *Firmicutes* as an example, its relative abundance changes at different time points were consistent with *Clostridia*, *Clostridiales*, and *Ruminococcaceae* (Fig. 2A) and showed their influence on the similarity of microbiota structure based on age. The microbial taxa (only at the family and genus levels) with the same change trends in relative abundance with age are shown in Fig. S3, and the representative genera of the corresponding family level were evident. As shown on the *x* axis, variations in relative abundance were also periodic, which were divided into day 3, days 7 to 14, days 21 to 28, and days 35 to 42. Here, the main effects of changes at different taxonomic levels on the microbiota structure have been explained longitudinally.

10.1128/msphere.00614-22.3 FIG S3 Microbial taxa at the family and genus levels with the same change trends in relative abundance with age. The heatmap color (blue to red, corresponding to low to high) represents the row z-score of the relative abundance values. The time points were clustered along the *x* axis based on their similarity across the degree of color. The *y* axis was clustered according to the microbiota with taxonomic affiliation. Download FIG S3, TIF file, 13.3 MB. Copyright © 2023 Yin et al. 2023 Yin et al. https://creativecommons.org/licenses/by/4.0/ This content is distributed under the terms of the Creative Commons Attribution 4.0 International license.

The linear discriminant analysis effect size (LEfSe) analysis showed biomarkers at various time points (Fig. 2B). The biomarkers of day 3 had two taxonomic branches, one was *Erysipelotrichi*, *Erysipelotrichale*s, *Erysipelotrichaeae*, and *Erysipelotrichae_Clostridium* (*P < *0.01), and the other was *Proteobacteria*, *Gammaproteobacteria*, *Enterobacteriales*, *Enterobacteriaceae*, and *Shigella* (*P < *0.01). In addition, *Melissacoccus* and *Pediococcus* of *Lactobacillales* and [*Ruminococcus*] of *Lachnospiraceae* were biomarkers (*P < *0.01). *Tenericutes*, *Mollicutes*, *Anaerotruncus*, and *Ruminococcus* were biomarkers of day 7 (*P < *0.01). The biomarkers of day 14 were *Bacillales*, *Bacillaceae*, *Dorea*, and *RF39* (*P < *0.05). The biomarkers of day 21 had two taxonomic branches, one was *Clostridia*, *Clostridiales*, and *Ruminococcaceae* (*P < *0.01), and the other was *Cyanobacteria*, *4C0d_2*, and *YS2* (*P < *0.01). The biomarkers of day 35 included multiple taxonomic branches, but the most significant were *Bacteroidetes*, *Bacteroidia*, *Bacteroidales*, *Bacteroidaceae*, and *Bacteroides* (*P < *0.01). Some microbial taxa from *Bacteroidales* were the primary biomarkers on day 42, such as [*Odoribacteraceae*], *Odoribacter*, *Porphyronadaceae*, and *Parabacteroides* (*P < *0.01). Note that biomarkers were not available on day 28, which may indicate that day 28 was a transition point for succession.

### Prediction of the nutrient metabolic function of the microbiota.

**FIG 3:**
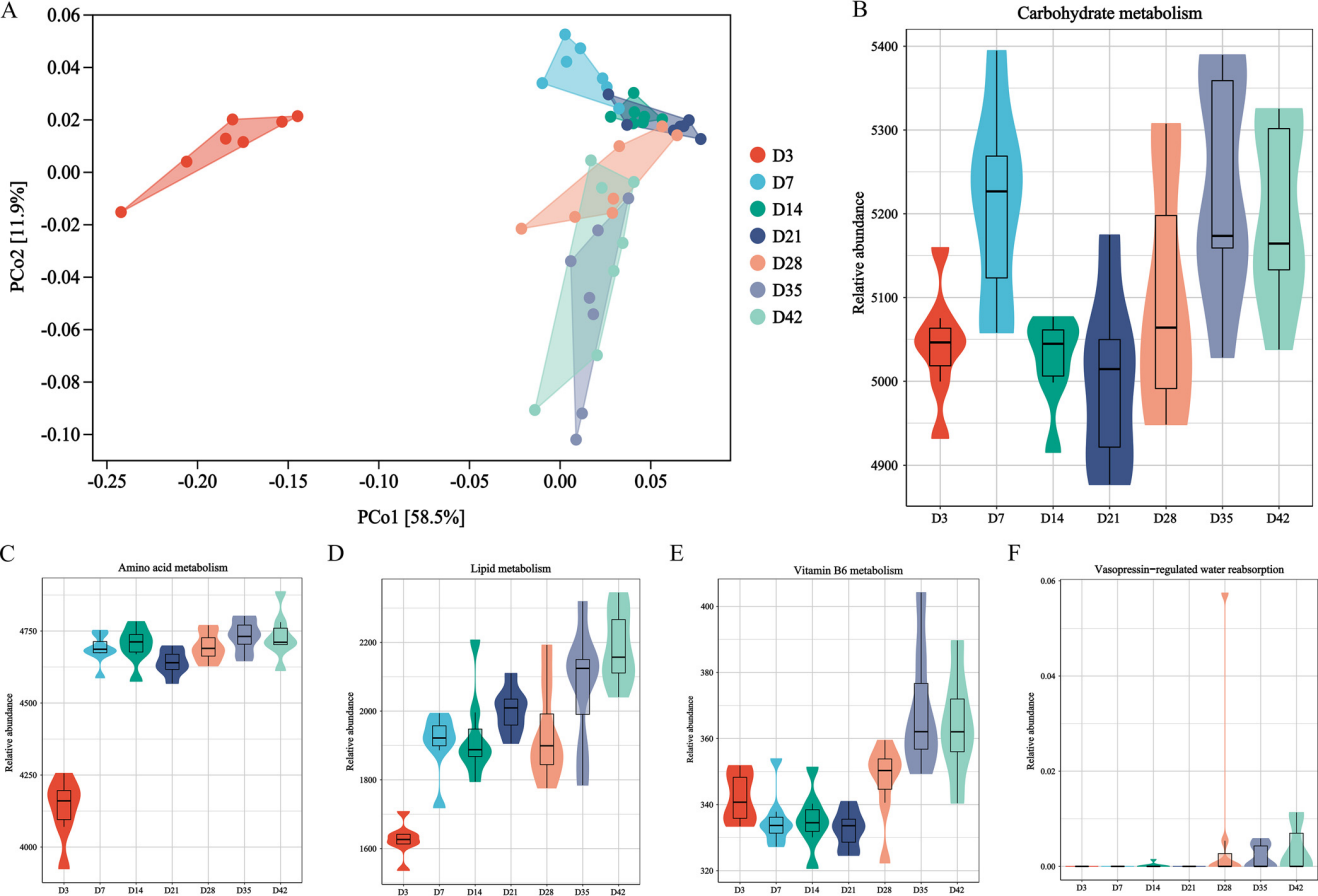
Functional prediction was based on 16S rRNA gene taxonomic inference in PICRUST2 using the Kyoto Encyclopedia of Genes and Genomes (KEGG) database. (A) PCoA of the KEGG Orthology (KO) genes. A Bray-Curtis distance matrix combined with PCoA was used for functional difference analysis. (B to D) The pathways of carbohydrate metabolism, amino acid metabolism, and lipid metabolism are based on KEGG level 2. (E and F) The pathways of vitamin B_6_ metabolism and vasopressin-regulated water reabsorption are based on KEGG level 3.

PICRUSt 2 was used to predict the functions of the cecal microbiota and to analyze levels 2 and 3 of the Kyoto Encyclopedia of Genes and Genomes (KEGG) pathways. The PCoA diagram showed that the microbiota function on day 3 was different from other time points (Fig. 3A). Based on the second characteristic value (Y-axis), it showed the phased changes of microbiota with age (Fig. 3A).

The KEGG level 2 pathways were revealed in Fig. S4. The cecal microbiota was mainly involved in metabolism, genetic information processing, environmental information processing, and cellular processes (see Fig. S4). Especially in the metabolic pathway, the microbiota mainly participates in carbohydrate metabolism, amino acid metabolism, lipid metabolism, and metabolism of cofactors and vitamins (see Fig. S4). The relative abundance of the carbohydrate metabolic pathway was the highest among all metabolic pathways and reached the highest level on day 35 (Fig. 3B). The relative abundance of the amino acid metabolic pathway increased with age and reached the highest level on day 42 (*P < *0.01, Fig. 3C). Similarly, the relative abundance of lipid metabolism pathway on days 35 or 42 was significantly higher than that on day 3 (*P < *0.01, Fig. 3D). In combination with the nutrient metabolic function of the microbiota and cecum, the changes in vitamin B metabolism and cecum participation in water reabsorption were noted. As shown in Fig. 3E, the relative abundance of vitamin B_6_ metabolic pathway gradually decreases with age, and that on day 21 was significantly lower than that on days 35 or 42 (*P < *0.01). The relative abundance of vasopressin-regulated water reabsorption pathway gradually increased with age (Fig. 3F). *P* values of the above KEGG pathways between any two time points are summarized in Table S3.

10.1128/msphere.00614-22.4 FIG S4 Pathways in the KEGG level 2. Download FIG S4, TIF file, 4.5 MB. Copyright © 2023 Yin et al. 2023 Yin et al. https://creativecommons.org/licenses/by/4.0/ This content is distributed under the terms of the Creative Commons Attribution 4.0 International license.

10.1128/msphere.00614-22.7 TABLE S3 Summary of *P* values of KEGG pathways between any two time points. Download Table S3, PDF file, 0.07 MB. Copyright © 2023 Yin et al. 2023 Yin et al. https://creativecommons.org/licenses/by/4.0/ This content is distributed under the terms of the Creative Commons Attribution 4.0 International license.

### Determination of relevant indicators of nutrient metabolism.

Combined with the metabolic processes of nutrients in the microbiota and gut and reference to the prediction of the KEGG pathways, the main indicators of nutrient-dependent regulation of metabolism (such as intestinal digestion, mucosal transport, blood circulation, and excretion processes) were measured.

### (i) Morphology of the cecum.

**FIG 4:**
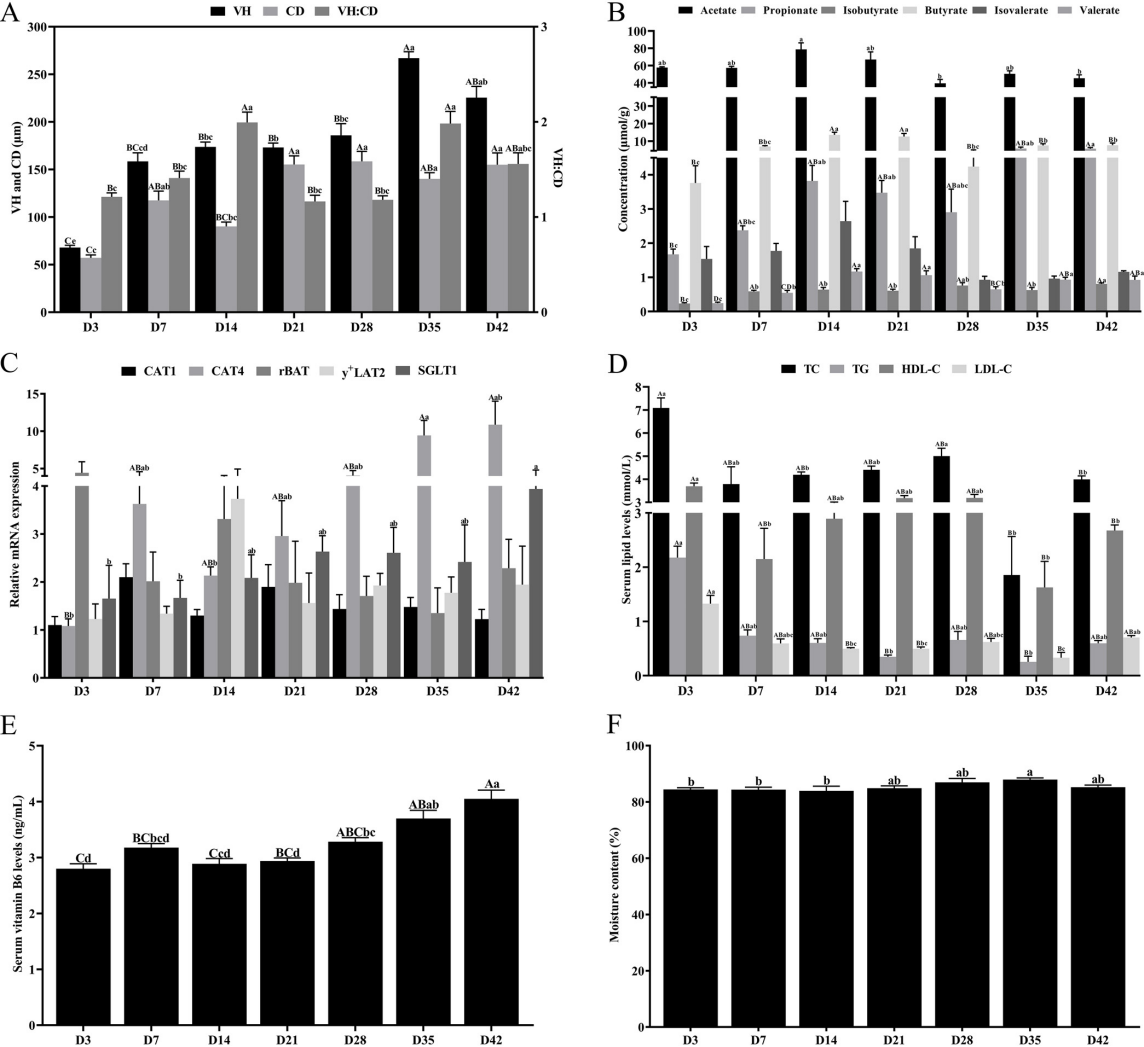
Determination of cecum morphology, SCFAs, glucose and amino acid transporters, serum lipid metabolites, serum VB6, and moisture of contents. (A) VH, CD, and VH:CD of the cecum. (B) Cecal concentrations of acetate, propionate, isobutyrate, butyrate, isovalerate, and valerate. (C) mRNA expression of rBAT, CAT1, CAT4, y^+^LAT2, and SGLT1 in the cecal mucosa. (D) Serum levels of TG, TC, HDL-C, and LDL-C. (E) Serum VB6 levels. (F) Moisture content of cecal contents. Abbreviations: SCFAs, short-chain fatty acids; VH, villus height; CD, crypt depth; VH:CD, villus height/crypt depth ratio; SGLT1, sodium-glucose cotransporters 1; y^+^LAT2, y^+^L amino acid transporter-2; rBAT, related to b^0,+^, neutral and basic amino acid transport protein; CAT1, cationic amino acid transporter 1; CAT4, cationic amino acid transporter 4; TG, triglycerides; TC, total cholesterol; HDL-C, high-density lipoprotein cholesterol; LDL-C, low-density lipoprotein cholesterol; VB6, vitamin B_6_. Significantly different values are indicated by different letters (lowercase letters, *P < *0.05; uppercase letters, *P < *0.01).

Villus height (VH) and crypt depth (CD) were the lowest on day 3 and increased with age, reaching the maximum on days 35 or 42 (*P < *0.01, Fig. 4A). The VH/CD ratio reached its maximum on days 14 or 35 (Fig. 4A).

### (ii) SCFAs of cecal contents.

SCFAs were mainly composed of acetate, butyrate, and propionate, and their concentrations gradually increased with age (Fig. 4B). The acetate concentration on day 14 was the highest. The propionate concentration was the lowest on day 3 and the highest on day 42 (*P < *0.01, Fig. 4B). The changing trend of isobutyrate concentration was similar to that of propionate, which was significantly lower on day 3 than on day 42 (*P < *0.01, Fig. 4B). The concentrations of butyrate and valerate on days 14 or 21 were significantly higher than those on day 3 (*P < *0.01, Fig. 4B).

### (iii) Nutrient transporters in cecal mucosa.

The mRNA levels of CAT1, rBAT, and y^+^LAT2 were no significant difference at different time points. Notably, the rBAT mRNA level was overexpressed on day 3 (Fig. 4C). The CAT4 mRNA level was the lowest on day 3 and significantly increased on day 35 (*P < *0.01, Fig. 4C). The mRNA level of SGLT1 gradually increased with age and was significantly expressed on day 42 (*P < *0.05, Fig. 4C).

### (iv) Lipid metabolites in serum.

It was worth noting that the levels of total cholesterol (TC), triglycerides (TG), high-density lipoprotein cholesterol (HDL-C), and low-density lipoprotein cholesterol (LDL-C) in serum were the highest on day 3 and the lowest on day 35 (*P < *0.01, Fig. 4D).

### (v) Serum VB6 levels.

Serum VB6 levels were low between day 3 and 21, and significantly higher on day 42 than the above periods (*P < *0.01, Fig. 4E).

### (vi) Moisture content of cecal contents.

The moisture content of cecal contents was over 80%, and highest on day 35 (*P < *0.05, Fig. 4F).

### The potential links between microbial succession and nutrient metabolism.

**FIG 5:**
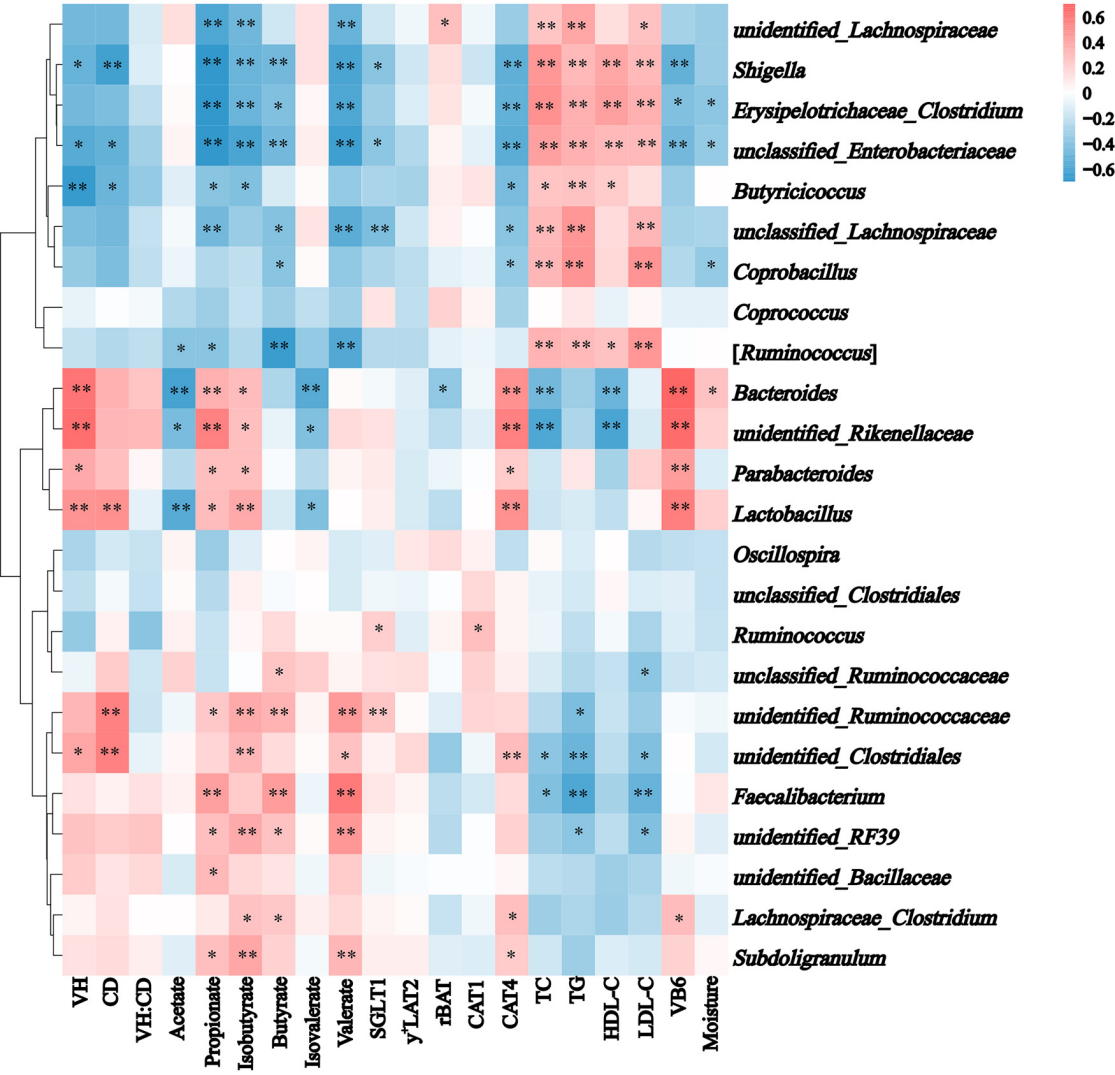
Heatmap showing Spearman correlations between predominant genera in the cecum and cecum morphology, SCFAs, glucose, and amino acid transporters, serum lipid metabolites, serum VB6, and moisture of contents. Red indicates a positive correlation; blue indicates a negative correlation. Abbreviations: SCFAs, short-chain fatty acids; VH, villus height; CD, crypt depth; VH:CD, villus height: crypt depth; SGLT1, sodium-glucose cotransporters 1; y^+^LAT2, y^+^L amino acid transporter-2; rBAT, related to b^0,+^, neutral and basic amino acid transport protein; CAT1, cationic amino acid transporter 1; CAT4, cationic amino acid transporter 4; TG, triglycerides; TC, total cholesterol; HDL-C, high-density lipoprotein cholesterol; LDL-C, low-density lipoprotein cholesterol; VB6, vitamin B_6_. *, 0.01 < *P ≤ *0.05; **, *P ≤ *0.01.

Combined with analysis of the microbiota structure at the genus level (Fig. 1C), a total of 24 genera (the shared and dominant genera) were used for Spearman’s correlation analysis. Overall, more than half of the proportion of correlations were significant (Fig. 5). (i) *Bacteroides*, *unidentified_Rikenellaceae*, *Lactobacillus*, and *Butyricicoccus* were significantly correlated with VH (*P < *0.01), and *Lactobacillus*, *unidentified_Ruminococcaceae*, and *unidentified_Clostridiales* were positively correlated with CD (*P < *0.01). (ii) *Bacteroides* and *Lactobacillus* were negatively correlated with acetate concentrations (*P < *0.01). *Bacteroides*, *unidentified_Rikenellaceae*, *unclassified_Lachnospiraceae*, *unidentified_Lachnospiraceae*, *Faecalibacterium*, *Erysipelotrichaceae_Clostridium*, *Shigella*, and *unclassified_Enterobacteriaceae* were significantly correlated with propionate concentrations (*P < *0.01). *Lactobacillus*, *Erysipelotrichaceae_Clostridium*, *Shigella*, *unclassified_Enterobacteriaceae*, and *unidentified_RF39* were significantly correlated with isobutyrate concentrations (*P < *0.01). [*Ruminococcus*], *Faecalibacterium*, *unidentified_Ruminococcaceae*, *Shigella*, and *unclassified_Enterobacteriaceae* were significantly correlated with butyrate concentrations (*P < *0.01). *Bacteroides* were negatively correlated with isovalerate concentrations (*P < *0.01). [*Ruminococcus*], *unclassified_Lachnospiraceae*, *unidentified_Lachnospiraceae*, *Faecalibacterium*, *unidentified_Ruminococcaceae*, *Erysipelotrichaceae_Clostridium*, *Shigella*, *unclassified_Enterobacteriaceae*, and *unidentified_RF39* were significantly correlated with valerate concentrations (*P < *0.01). (iii) *unclassified_Lachnospiraceae*, *Ruminococcus*, *unidentified_Ruminococcaceae*, *Shigella*, and *unclassified_Enterobacteriaceae* were significantly correlated with SGLT1 expression (*P < *0.05). *Bacteroides* and *unidentified_Lachnospiraceae* were significantly correlated with rBAT expression (*P < *0.05). *Ruminococcus* was positively correlated with CAT1 expression (*P < *0.05). *Bacteroides*, *unidentified_Rikenellaceae*, *Lactobacillus*, *unidentified_Clostridiales*, *Shigella*, and *unclassified_Enterobacteriaceae* were significantly correlated with CAT4 expression (*P < *0.01). (iv) [*Ruminococcus*], *unclassified_Lachnospiraceae*, *Erysipelotrichaceae_Clostridium*, *Coprobacillus*, *Shigella*, and *unclassified_Enterobacteriaceae* were positively correlated with TC, TG, and LDL-C levels (*P < *0.01). *Erysipelotrichaceae_Clostridium*, *Shigella*, and *unclassified_Enterobacteriaceae* were positively correlated with HDL-C levels (*P < *0.01). (v) *Bacteroides*, *Parabacteroides*, *Unclassified_Rikenellaceae*, *Lactobacillus*, *Shigella*, and *unclassified_Enterobacteriaceae* were significantly correlated serum VB6 levels (*P < *0.01). (vi) *Bacteroides*, *Erysipelotrichaceae_Clostridium*, *Coprobacillus*, and *unclassified_Enterobacteriaceae* were significantly correlated with the moisture content of cecal contents (*P < *0.05).

## DISCUSSION

We sought to elucidate trends in the development of the cecal microbiota in broiler chickens during the commercial production cycle and how they associate with host nutrient metabolic functions over time. The results showed that age was the major factor influencing the composition of the cecal microbiota in poultry flocks, and this was consistent with most studies (28–30). At each time point, there was a unique microbiota structure, which was different from relevant studies (15, 17, 31). Furthermore, whether there is an optimal time for microecological regulation or when to take action is a hot spot in poultry research. It has been pointed out that the best time to intervene in the gut microbiome of broiler chickens was before and after the day of hatch (32), days 14 to 21 (33), or days 20 to 30 (17). The other two intervention periods were the preparation period for slaughter transport and periods of disturbance or imbalance in the gut microbiome (34). Regulatory windows appeared at critical stages in the succession of the gut microbiota, and their common feature was the dynamic variability in the development of the gut microbiota. In this study, the sampling time points interval was taken as weeks of age to consider the adjustment of dietary composition with age and to refer to the above intervention periods. LEfSe and taxonomic composition analysis provided potential biomarkers for each time point, KEGG pathways analysis revealed that metabolism was the main pathway, while correlation analysis further establishes the association between dominant genera and nutrient metabolic indicators, which was further evidence and development of the above regulatory periods and the interaction between microbial metabolism and host nutrition.

### Succession of the cecal microbiota with age.

PCoA showed that the microbiota structure on days 3 and 7 was significantly different from other time points, but the difference was no longer significant from day 14. Relevant research also showed that the first two stages of microbial succession in broiler chickens were days 1 to 3 and days 4 to 9, which may be related to the characteristics of rapid intestinal development (35, 36). The dominant phyla, such as *Firmicutes*, *Bacteroidetes*, and *Proteobacteria*, were not only similar to the composition of the ordinary chicken microbial database (37) but also the primary category of gastrointestinal microbiota in poultry (38, 39). The *Firmicutes*’ dominant position throughout the production period may be to meet the need for intestinal development or host energy (40). It has been reported that the relative abundance of *Firmicutes* was highest at week 2 and then decreased with age, and the proportion of *Bacteroidetes* was relatively stable (41). However, the relative abundance of *Firmicutes* on day 14 continued to increase with age and may represent an important step in the development and maturation of the microbiota in this study. Related studies had shown that Gram-positive bacteria, such as *Lachnospiraceae* and *Ruminococcaceae*, were dominant in week 2 (12), and this basic feature continued in this study. Studies suggested that *Bacteroidaceae* were the colonizers of *Bacteroidetes* in the later period of poultry production (12), and stable colonization of *Bacteroidetes* began on day 28 and reached a certain proportion with age in this study. *Bacteroides* used complex polysaccharides to produce propionate and butyrate (42). The *Firmicutes*/*Bacteroidetes* ratio gradually decreased with age, which may indicate that the energy supply gradually transitioned from the butyrate-producing pathway of *Firmicutes* to the balance of the two acid-producing pathways. Predominant colonization by *Proteobacteria* (e.g., *Enterobacteriaceae*) on day 3 in this study and early life in other studies may be due to low post-hatch microbial diversity and high susceptibility to environmental microbes (33, 43). Pathogens such as Escherichia coli dominate the cecum within the first week of life (12). Correspondingly, in this study, *Shigella* was colonized as a conditional pathogen on day 3.

We analyzed trend associations in microbiota dynamics at various taxonomic levels with age. These dominant phyla, like *Firmicutes*, significantly affect the structural stability of the microbiota (40). Interestingly, it corresponded to the dynamic change tendency for *Clostridia*, *Clostridiales*, and *Ruminococcus* throughout the production period. The relative stability of microbial systems appears to be determined by these microbial taxa that were dominantly colonized in their taxonomic composition (44). To our minds, distinct taxonomically dominant microbiota (endogenous factors) are progressively established and colonized with stability over age (external causes) in poultry.

### Succession of dominant genera according to the stages of production.

At the genus level, we focus on the succession of several major genera. Some taxa were always present in the chicken gut (44), although there were numerous differences among the chicken microbiota (45). Identifying these common and dominant taxa and further exploring their functions will lead to a better understanding of the microbial ecology of the chicken gut. Comparison to public databases revealed common dominant genera such as *Clostridium*, *Ruminococcus*, and *Bacteroides* (37), as well as unique-dominant genera such as *Faecalibacterium*, [*Rumococcus*], and *Oscillospira* in this study. The above results suggested that individual differences affect the composition of the microbiota. It should be noted that the dominant genera with high relative abundance, such as *unidentified_Clostridiales*, *unidentified_Lachnospiraceae*, and *unidentified_Ruminococcaceae*, require further identification of the taxonomic state due to limited sequencing technology or to not being studied. However, these three genera were also included in the subsequent analysis because they had a greater impact on the structure of the microbiota.

The mechanism driving the development of the cecal microbiota seems difficult to clarify, but we wanted to try to analyze the processes of gut microbiota development in conjunction with the production stages. Each stage should have its goal of nutritional regulation, which should be different and targeted. This was one of the reasons why we analyze and discuss the microbial community structure at all-time points. First, the structure of the microbiota changed rapidly during the brooding period (days 0 to 7). The cecal microbiota on day 3 after the day of hatch had a unique structure from that at hatching, suggesting that the structure of the early microbial community was transient (15). On the other hand, we found that the membership of the shared-dominant genera was similar to that on day 3, suggesting that the microbiota structure of day 3 also laid the foundation for the entire production period. The above points to the beginning of life as one of the best time points for microbiological regulation. *Shigella* of *Enterobacteriaceae* dominated on day 3, which was similar to the related studies (43). Day 7, as a transition time point for early microbiome development, was very different from day 3. *Ruminococcus* was representative on day 7, producing SCFAs through glucose metabolism and cellulose degradation (46). At the end of the transition period on day 14, *Faecalibacterium* began to dominate and secrete enzymes required for butyrate production (40). During the period of rapid bone growth (days 14 to 21), the structure of the microbiota was relatively stable, consistent with the recent study (31). During the period of feed change (days 21 to 28), the microbiota community was adjusted, which may be caused by the changes in nutritional level. The high abundance of *unidentified_Clostridiales* of *Clostridiales* and *unidentified_Ruminococcaceae* of *Ruminococcaceae* may reflect changes caused by the dietary. Throughout rapid weight gain (days 28 to 42) (15), the structure of the microbiota has changed considerably. Such as the relative abundance of *Bacteroides* significantly increased on day 35 and may represent an increase in the propionate production (40). Generally, the microbiota was characterized by a progressive succession with age.

### The cecal microbiome is intimately associated with host metabolism.

The diverse microbial communities had a broad metabolic spectrum, which played a key role in regulating host metabolism and health by shaping the biochemical characteristics of the diet (3). The development and stability of the microbiome were directed by the host (14). This inter-relationship is also reflected in the effect of the microbiome on the host, such as when the regulation of intestinal morphology, in turn, affects the digestion and absorption of nutrients. For example, *Lactobacillus* contributed to the development of intestinal morphology in broiler chickens (47), and we also found that *Lactobacillus* was positively correlated with VH and CD.

Rapid weight gain in early life requires efficient nutrient absorption, and SCFAs provide the host with high-quality energy (12). It has been reported that the *Ruminococcaceae* of *Firmicutes* were the major SCFA-producing microbiota in the gut (48), and we found that *Faecalibacterium* of *Ruminococcaceae* was positively correlated with butyrate concentrations. *Lachnospiraceae* can promote gut development and health by degrading plant fiber to SCFAs (49). Nevertheless, we found that [*Ruminococcus*] of *Lachnospiraceae* was negatively correlated with butyrate concentrations. On the one hand, the genus may be unclassified, and its function is unknown; on the other hand, SCFAs may vary with dietary composition or feeding system (50). Ruminococcus gnavus contains degrading enzymes needed for the catabolism of amino acids (51), and *Ruminococcus* had a positive correlation with the expression of CAT1 in our findings. Ruminococcus albus could secrete β-glucosidase and hydrolyze cellobiose and cellooligosaccharides into glucose (52). Interestingly, there was a positive correlation between *Ruminococcus* and SGLT1 expression. Avian cecum contains large amounts of micronutrients produced by microbiota, such as B vitamins (13). *Lactobacillus* played a key role in the production of B vitamins (53), and *Bacteroides* captured vitamin B_12_ through lipoproteins exposed on the cell surface (54). We also found a significant positive association between the above bacteria and serum VB6 levels.

Gut microbes were closely related to the occurrence and development of various nutritional metabolic diseases (55). Lipid deposition caused by disorderly fat metabolism reduces meat quality and feed utilization in poultry production. Some members of the *Erysipelotrichaceae* lacked partial genes related to fatty acid biosynthesis (56), the abundance of *Erysipelotrichaceae* was significantly decreased in a hamster model in which the extract improved hypercholesterolemia (57), and *Erysipelotrichaceae_Clostridium* had significant positive correlations with the four lipid metabolites in this study. The cecum maintains the balance of water and electrolytes in poultry, but enteritis caused by bacterial infection can lead to decreased water and salt absorption and excess water in the feces contamination of the environment. Enterotoxins secreted by Bacteroides fragilis had been reported to disrupt tight-junction barriers and cause diarrhea (58), and we also found a positive correlation between *Bacteroides* and the moisture content of cecal contents. These correlation analyses may provide ideas regarding microecological regulation to solve industrial problems such as poultry lipid metabolic diseases and watery stools.

To better understand the mechanisms driving microbial development and to identify potential probiotic candidates associated with nutrient metabolism, the 16S rRNA gene sequences at the genus, species, and strain levels must be further elucidated. Although this study found some correlation between the nutrient metabolic function of microbiota or hosts and the composition of cecal dominant microbiota, certain correlations may not be entirely consistent with some relevant studies. However, all of these still need to be verified, especially the actual functions of related probiotic candidates in poultry. In addition, more sample sizes and different farms, with more pronounced differences in feed nutrient levels or nutrient metabolic function, may show more associations between microbiota composition and nutrient metabolism.

## MATERIALS AND METHODS

### Experimental design and sampling.

All experimental protocols have been approved by Northwest A&F University’s Institutional Animal Care and Use Committee in China. The protocols of this study were specifically approved under protocol NWAFAC1008. A completely randomized design was used in this study. A total of 280 1-day-old Arbor Acres commercial broiler chicks were randomly allotted to 7 replicates with 40 birds per replicate. The basal diet (see Table S4) was fed throughout the trial period (6 weeks). Samples were collected on days 3, 7, 14, 21, 28, 35, and 42. On day 3, three birds with body weights close to the mean body weight of each replicate were randomly selected. On day 7, two birds were selected from each replicate at random. On days 14, 21, 28, 35, and 42, one bird was randomly selected from each replicate for sampling. A total of 70 birds were selected in seven replicates at seven time points. Fresh cecal contents were collected, and a portion of the contents was immediately used for the determination of moisture content. The remainder was immediately frozen in liquid nitrogen and then stored at −80°C for 16S rRNA sequencing and determination of SCFA concentrations. To reduce the individual differences and experimental errors caused by fewer cecal contents, and to accurately measure the above three indicators, the samples of day 3 (equal amounts of cecal contents from three birds in each replicate were pooled into one sample) and day 7 (equal amounts of cecal contents from two birds in each replicate were pooled into one sample) were pooled. Thus, on days 3 and 7, one mixed sample collection was regarded as an experimental unit for determining moisture content, microbiota, and SCFAs. And the replicate of one bird used for sample collection was regarded as an experimental unit to determine the other indicators. For all other time points (days 14, 21, 28, 35, and 42), no pooled sampling was performed. An ~1-cm cecal segment from the proximal end of the cecum was collected and fixed in 4% paraformaldehyde and stored at 4°C. The mucosa in the distal region of the cecum, ~3 cm from the ileocecal junction, was scraped and stored at −80°C. Blood samples (~3 mL) were collected from the jugular vein, and the serum was stored at −80°C.

10.1128/msphere.00614-22.8 TABLE S4 Composition and nutrient levels of basal diet (as-fed basis [%]). Download Table S4, PDF file, 0.09 MB. Copyright © 2023 Yin et al. 2023 Yin et al. https://creativecommons.org/licenses/by/4.0/ This content is distributed under the terms of the Creative Commons Attribution 4.0 International license.

### Microbial 16S rRNA sequencing.

Total bacterial genomic DNA samples were extracted using an Omega soil DNA kit (M5635-02) (Omega Bio-Tek, Norcross, GA). PCR amplification of the bacterial 16S rRNA genes V3-V4 region was performed using the forward primer 338F (5′-ACTCCTACGGGAGGCAGCA-3′) and the reverse primer 806R (5′-GGACTACHVGGGTWTCTAAT-3′). Sample-specific 7-bp barcodes have been incorporated into the primers for multiplex sequencing. The PCR components contained 5 μL of buffer, 0.25 μL of Fast PFU DNA polymerase, 2 μL of deoxynucleoside triphosphates, 1 μL of each forward and reverse primer, 1 μL of DNA template, and 14.75 μL of ddH_2_O. Thermal cycling consisted of initial denaturation at 98°C for 5 min, followed by 25 cycles consisting of denaturation at 98°C for 30 s, annealing at 53°C for 30 s, and extension at 72°C for 45 s, with a final extension of 5 min at 72°C. PCR amplicons were purified with Vazyme VAHTSTM DNA Clean Beads (Vazyme, Nanjing, China) and quantified using the Quant-iT PicoGreen dsDNA assay kit (Invitrogen, Carlsbad, CA). After the individual quantification step, amplicons were pooled in equal amounts, and pair-end 2 × 250 bp sequencing was performed using the Illumina NovaSeq platform with NovaSeq 6000 SP reagent kit (500 cycles) at Shanghai Personal Biotechnology Co., Ltd. (Shanghai, China).

Microbiome bioinformatics was performed with QIIME2 2019.4 (59) with slight modifications according to the official tutorials. Raw sequence data were demultiplexed using the demux plugin (60), quality filtered, denoised, and merged, and chimeras were removed using the DADA2 plugin (61). Nonsingleton amplicon sequence variants (ASVs) were aligned with mafft (62) and used to construct a phylogeny with fasttree2 (63). Alpha-diversity metrics (Chao1, Shannon, Pielou’s evenness, and Good’s coverage), and beta-diversity metrics (weighted UniFrac) (64) were estimated using the diversity plugin. Taxonomy was assigned to ASVs using the classify-sklearn naive Bayes taxonomy classifier in the feature-classifier plugin (65) against the Greengenes database (v13.8) (66).

Sequence data analyses were mainly performed with QIIME2 and R packages (v3.2.0). ASV-level alpha-diversity indices were calculated using the ASV table and visualized as box plots. ASV-level ranked abundance curves were generated to compare the richness and evenness of ASVs among samples. Beta-diversity analysis was conducted to examine the structural variation of microbial communities across samples using UniFrac distance metrics (64, 67) and visualized via PCoA. The significance of the differentiation of microbiota structure among groups was evaluated by PERMANOVA (68) and ANOSIM (69, 70). LEfSe was performed to detect differentially abundant taxa between groups using the default parameters (71). Nested stratified k-fold cross validation was used for automated hyperparameter optimization and sample prediction, and the number of k-fold cross-validations was set to 5. Microbial functions were predicted by PICRUSt2 (72) upon KEGG (https://www.kegg.jp/) databases. Raw sequences were deposited in the NCBI Sequence Read Archive (SRA) under accession number PRJNA937609.

### Cecal histomorphology analysis.

Paraformaldehyde-fixed samples were prepared according to the conventional paraffin-embedding techniques. Paraffin sections (5 mm) were cut and stained using hematoxylin and eosin (H&E). Villus height (VH) and crypt depth (CD) were measured from five appearance-intact villi with Image-Pro Plus 6.0 (Media Cybernetics, Inc., Rockville, MD). The VH/CD ratio (VH:CD) was then calculated.

### Determination of SCFAs.

Cecal contents (~0.5 g dissolved in 1.5 mL of sterile water until completely dissolved) were centrifuged at 13,000 × *g* for 10 min at 4°C and then assayed as described previously (73). Briefly, each 1.5 mL of supernatant was mixed with 0.2 mL of metaphosphoric acid, followed by centrifugation at 10,000 × *g* for 15 min at 4°C. Then, 1 mL of supernatant was mixed with 200 μL of crotonic acid at 4°C for 1 h, and the sample was filtered with a filter membrane (0.45-μm pore size). The SCFAs (acetate, propionate, butyrate, isobutyrate, valerate, and isovalerate) were quantified using an Agilent 7820A gas chromatography system.

### Quantitative real-time PCR.

The total RNA was extracted by TRIzol reagent (TaKaRa, Dalian, China) from cecal mucosa and stored at −80°C. The cDNA was obtained by reverse transcription using a Primer Script RT kit (TaKaRa) and stored at −20°C. The SYBR Premix *Ex Taq* kit (TaKaRa) was used for determination on the IQ5 (Bio-Rad, Hercules, CA). The PCR conditions were 95°C for 30 s; followed by 45 cycles of 95°C for 5 s, 60°C for the 30 s, and 72°C for 30 s; followed by 72°C for 5 min. All samples were run in triplicate, and the average cycle threshold (*C_T_*) was calculated using the 2^−ΔΔ^*^CT^* method (74). The relative expression was calculated based on the β-action expression. The primer sequences used for quantitative real-time PCR analysis are listed in Table S5, including the cationic amino acid transporters CAT1, CAT4, rBAT, and y^+^LAT2 and the sodium-glucose cotransporter SGLT1.

10.1128/msphere.00614-22.9 TABLE S5 Sequences of primers used for the quantitative real-time PCR analysis. Download Table S5, PDF file, 0.08 MB. Copyright © 2023 Yin et al. 2023 Yin et al. https://creativecommons.org/licenses/by/4.0/ This content is distributed under the terms of the Creative Commons Attribution 4.0 International license.

### Serum analysis.

Serum was collected by centrifugation at 3,000 g for 15 min at 4°C, aliquoted, and stored at −80°C until analysis. TG, TC, HDL-C, and LDL-C were analyzed at the Yangling Demonstration Zone Hospital (Yangling, Shaanxi, China) using a fully automatic biochemical analyzer (ADVIA2400; Siemens, Berlin, Germany). Vitamin B_6_ (VB6) was analyzed using an enzyme-linked immunosorbent assay (catalog no. RJ29885; Shanghai Renjie Biotechnology Co., Ltd., Shanghai, China).

### Moisture content.

The moisture content was determined by the oven method (75). An empty porcelain crucible was oven-dried to a constant weight at 105°C, allowed to cool in a desiccator, and weighed (W_1_). The cecal content sample (1.0 g) was weighed (W_2_) in the crucible and oven-dried at 105°C until it attained a constant weight. The crucible containing the sample of the cecal contents was allowed to cool in a desiccator and the weight (W_3_) was measured. The moisture content was calculated as a percentage: [(W_2_ – W_3_)/(W_2_ – W_1_)] × 100%.

### Statistical analysis.

SPSS 22.0 software (IBM, Chicago, IL) was used to analyze the statistical data. When the data were normally distributed with homogeneity of variance, significance was determined by one-way analysis of variance (ANOVA), followed by Duncan’s multiple range test. Welch’s ANOVA was used when the normally distributed datasets have unequal variances. If the data did not obey a normal distribution and the variance was not uniform, a Kruskal-Wallis test was used to analyze differences between groups. The Kruskal-Wallis test was used for pairwise comparison of microbiota composition (phylum and genus level) and KEGG pathways. Bar graphs were plotted by using GraphPad Prism 8.3.0 software. Results are shown as means ± the standard errors of the mean. A statistical test was considered significant when *P < *0.05. Correlations were analyzed using Spearman’s correlation procedure (two tailed), and *P < *0.05 was considered statistically significant.

### Data availability.

Raw sequences were deposited in the NCBI SRA under accession number PRJNA937609.
