# Supplementary figures and images for: Cecal Microbial Succession and Its Apparent Association with Nutrient Metabolism in Broiler Chickens
Source: mSphere. 2023 Apr 5;8(3):e00614-22. doi: 10.1128/msphere.00614-22 (PMC10286727; doi:10.1128/msphere.00614-22)

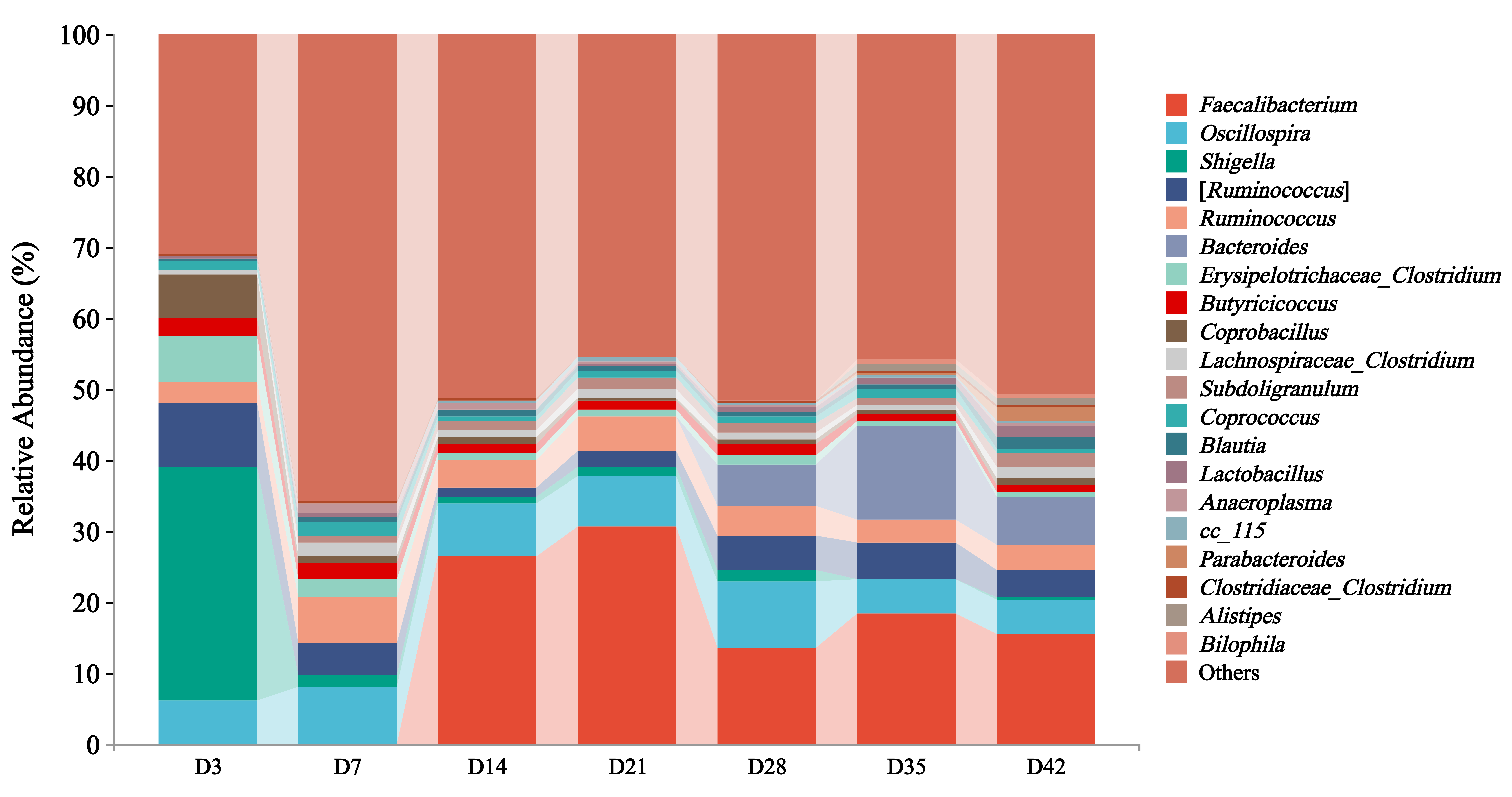

Supplement: FIG S2 [file msphere.00614-22-s0007.tif]
