# Supplementary material for: Cecal Microbial Succession and Its Apparent Association with Nutrient Metabolism in Broiler Chickens
Source: mSphere. 2023 Apr 5;8(3):e00614-22. doi: 10.1128/msphere.00614-22 (PMC10286727; doi:10.1128/msphere.00614-22)
Supplement: TABLE S2 [file msphere.00614-22-s0002.pdf]

Table S2 The Summary of *P*-values of genus between any two time points.

Genus	*P*-value between two groups																				
	AB	AC	AD	AE	AF	AG	BC	BD	BE	BF	BG	CD	CE	CF	CG	DE	DF	DG	EF	EG	FG
Faecalibacterium	>0.05	0.002	<0.001	>0.05	>0.05	>0.05	0.001	<0.001	>0.05	>0.05	>0.05	>0.05	>0.05	>0.05	>0.05	>0.05	>0.05	>0.05	>0.05	>0.05	>0.05
Oscillospira	>0.05	>0.05	>0.05	>0.05	>0.05	>0.05	>0.05	>0.05	>0.05	>0.05	>0.05	>0.05	>0.05	>0.05	>0.05	>0.05	>0.05	>0.05	0.027	>0.05	>0.05
Shigella	>0.05	>0.05	>0.05	>0.05	0.001	<0.001	>0.05	>0.05	>0.05	>0.05	>0.05	>0.05	>0.05	>0.05	>0.05	>0.05	>0.05	>0.05	>0.05	>0.05	>0.05
[*Ruminococcus*]	>0.05	<0.001	0.002	>0.05	>0.05	>0.05	>0.05	>0.05	>0.05	>0.05	>0.05	>0.05	>0.05	0.035	>0.05	>0.05	>0.05	>0.05	>0.05	>0.05	>0.05
Ruminococcus	0.002	>0.05	>0.05	>0.05	>0.05	>0.05	>0.05	>0.05	>0.05	0.024	>0.05	>0.05	>0.05	>0.05	>0.05	>0.05	>0.05	>0.05	>0.05	>0.05	>0.05
Bacteroides	>0.05	>0.05	>0.05	>0.05	<0.001	0.019	>0.05	>0.05	>0.05	0.005	>0.05	>0.05	>0.05	<0.001	0.019	>0.05	>0.05	>0.05	>0.05	>0.05	>0.05
Erysipelotrichaceae_Clostridium	>0.05	0.016	0.013	>0.05	0.001	0.002	>0.05	>0.05	>0.05	0.04	>0.05	>0.05	>0.05	>0.05	>0.05	>0.05	>0.05	>0.05	>0.05	>0.05	>0.05
Butyricicoccus	-	-	-	-	-	-	-	-	-	-	-	-	-	-	-	-	-	-	-	-	-
Coprobacillus	>0.05	>0.05	0.003	0.014	0.004	>0.05	>0.05	>0.05	>0.05	>0.05	>0.05	>0.05	>0.05	>0.05	>0.05	>0.05	>0.05	>0.05	>0.05	>0.05	>0.05
Lachnospiraceae_Clostridium	0.033	>0.05	>0.05	>0.05	>0.05	>0.05	>0.05	>0.05	>0.05	>0.05	>0.05	>0.05	>0.05	>0.05	>0.05	>0.05	>0.05	>0.05	>0.05	>0.05	>0.05
Subdoligranulum	>0.05	>0.05	0.004	0.035	>0.05	0.006	>0.05	>0.05	>0.05	>0.05	>0.05	>0.05	>0.05	>0.05	>0.05	>0.05	>0.05	>0.05	>0.05	>0.05	>0.05
Coprococcus	-	-	-	-	-	-	-	-	-	-	-	-	-	-	-	-	-	-	-	-	-
Blautia	-	-	-	-	-	-	-	-	-	-	-	-	-	-	-	-	-	-	-	-	-
Lactobacillus	>0.05	>0.05	>0.05	>0.05	>0.05	0.013	>0.05	>0.05	>0.05	>0.05	>0.05	>0.05	>0.05	0.022	0.001	>0.05	>0.05	>0.05	>0.05	>0.05	>0.05
Anaeroplasma	0.005	0.012	0.032	>0.05	>0.05	>0.05	>0.05	>0.05	>0.05	0.005	>0.05	>0.05	>0.05	0.012	>0.05	>0.05	0.032	>0.05	>0.05	>0.05	>0.05
cc_115	-	-	-	-	-	-	-	-	-	-	-	-	-	-	-	-	-	-	-	-	-
Parabacteroides	>0.05	>0.05	>0.05	>0.05	>0.05	0.002	>0.05	>0.05	>0.05	>0.05	0.029	>0.05	>0.05	>0.05	0.002	>0.05	>0.05	0.002	>0.05	0.002	0.04
Clostridiaceae_Clostridium	>0.05	>0.05	>0.05	>0.05	0.028	>0.05	>0.05	>0.05	>0.05	>0.05	>0.05	>0.05	>0.05	>0.05	>0.05	>0.05	>0.05	>0.05	>0.05	>0.05	>0.05
Alistipes	-	-	-	-	-	-	-	-	-	-	-	-	-	-	-	-	-	-	-	-	-
Bilophila	-	-	-	-	-	-	-	-	-	-	-	-	-	-	-	-	-	-	-	-	-

Note: A = day 3; B = day 7; C = day 14; D = day 21; E = day 28; F = day 35; G = day 42. - no significant difference between the groups.
