# Supplementary material for: Cecal Microbial Succession and Its Apparent Association with Nutrient Metabolism in Broiler Chickens
Source: mSphere. 2023 Apr 5;8(3):e00614-22. doi: 10.1128/msphere.00614-22 (PMC10286727; doi:10.1128/msphere.00614-22)
Supplement: TABLE S4 [file msphere.00614-22-s0004.pdf]

Table S4 Composition and nutrient levels of basal diet (as-fed basis, %).

Ingredients	Starter stage（days 1 to 21）	Grower stage（days 22 to 42）
Corn	57.10	63.51
Soybean meal（46%）	22.60	16.30
Distillers dried grains	5.00	5.00
Corn gluten meal（60%）	4.00	4.00
Cottonseed meal（45%）	3.00	4.00
Flour	2.50	—
Calcium hydrogen phosphate (powder)	1.83	1.24
Soy bean oil	1.20	2.30（lard）
Calcium powder	0.90	1.36
Lysine sulfate（70%）	0.84	1.10
Yellow bentonite	0.08	0.08
Salt	0.30	0.30
DL-Methionine（99%）	0.23	0.30
Threonine	0.13	0.21
Choline chloride（50%）	0.08	0.10
Vitamin premix[1]	0.05	0.05
Mineral premix[2]	0.15	0.15
Total	100	100
Nutritional level（%）		
Metabolizable energy (kcal/kg)	2,900	3,000
Crude protein %	21.00	19.00
Calcium %	0.90	0.90
Total phosphorus %	0.70	—
Available phosphorus %	0.45	0.35
Lysine %	1.30	1.30
Methionine %	0.56	0.60

[1] Supplied per kg of diet: (d 1 to 21) vitamin A, 11200 IU; vitamin D3, 3360 IU; vitamin E, 20 mg; vitamin K3, 4.0 mg; vitamin B1, 2.2 mg; vitamin B2, 7.28 mg; vitamin B6, 4.8 mg; (d 22 to 42) vitamin A, 8400 IU; vitamin D3, 2520 IU; vitamin E, 15.75 mg; vitamin K3, 3.0 mg; vitamin B1, 1.64 mg; vitamin B2, 5.46 mg; vitamin B6, 3.6 mg.

[2] Supplied per kg of diet: Cu, 8.4 mg; Fe, 54 mg; Zn, 49.5 mg; Mn, 70.5 mg.
