# Supplementary material for: Cecal Microbial Succession and Its Apparent Association with Nutrient Metabolism in Broiler Chickens
Source: mSphere. 2023 Apr 5;8(3):e00614-22. doi: 10.1128/msphere.00614-22 (PMC10286727; doi:10.1128/msphere.00614-22)
Supplement: TABLE S5 [file msphere.00614-22-s0005.pdf]

Table S5 Sequences of primers used for the quantitative real-time PCR analysis.

Primer name[1]	GenBank number	Sequences (5' to 3')[2]	Product length (bp)
rBAT	XM426125.1	F: CTACCAGGTCTACCCTCGTTC<br>R: TTCCCATAGACACTCACCCA	414
y^+LAT2	XM413988	F: CCTGATAGTAGGCAACAT<br>R: AGAACAAGGCAGAGTAGAG	581
CAT1	NW060243	F: ATTTGGTGCTCGTGTTCCT<br>R: TTGTAAATGTCCCGTTCAGTC	420
CAT4	XM424658	F: CTGGTGGGTAGTGACAAGA<br>R: TGCCGTAGCCAAAGTAGA	433
SGLT1	AJ236903	F: TGGTTGTTCTAGGATGGGTG<br>R: CAGTGACAGCATCTCGGAAG	489
β-action	L08165.1	F: GAGAAATTGTGCGTGACATCA<br>R: CCTGAACCTCTCATTGCCA	152

[1] SGLT1, sodium-glucose cotransporters 1; y^+LAT2, y^+L amino acid transporter-2; rBAT, related to $b^{0,+}$, neutral and basic amino acid transport protein; CAT1, cationic amino acid transporter 1; CAT4, cationic amino acid transporter 4;

[2] F, forward; R, reverse.
