# Supplementary material for: Cecal Microbial Succession and Its Apparent Association with Nutrient Metabolism in Broiler Chickens
Source: mSphere. 2023 Apr 5;8(3):e00614-22. doi: 10.1128/msphere.00614-22 (PMC10286727; doi:10.1128/msphere.00614-22)
Supplement: TABLE S3 [file msphere.00614-22-s0003.pdf]

Table S3 The Summary of *P*-values of KEGG pathways between any two time points.

Pathway	*P*-value between two groups																				
	AB	AC	AD	AE	AF	AG	BC	BD	BE	BF	BG	CD	CE	CF	CG	DE	DF	DG	EF	EG	FG
Carbohydrate metabolism	-	-	-	-	-	-	-	-	-	-	-	-	-	-	-	-	-	-	-	-	-
Amino acid metabolism	>0.05	0.015	>0.05	0.035	0.001	0.002	>0.05	>0.05	>0.05	>0.05	>0.05	>0.05	>0.05	>0.05	>0.05	>0.05	>0.05	>0.05	>0.05	>0.05	>0.05
Lipid metabolism	>0.05	>0.05	0.016	>0.05	0.002	<0.001	>0.05	>0.05	>0.05	>0.05	>0.05	>0.05	>0.05	>0.05	>0.05	>0.05	>0.05	>0.05	>0.05	>0.05	>0.05
Vitamin B6 metabolism	>0.05	>0.05	>0.05	>0.05	>0.05	>0.05	>0.05	>0.05	>0.05	>0.05	>0.05	>0.05	>0.05	>0.05	>0.05	>0.05	0.003	0.007	>0.05	>0.05	>0.05
Vasopressin-regulated water reabsorption	-	-	-	-	-	-	-	-	-	-	-	-	-	-	-	-	-	-	-	-	-

Note: A = day 3; B = day 7; C = day 14; D = day 21; E = day 28; F = day 35; G = day 42. - no significant difference between the groups.
